# Study on the Treatment of Refined Sugar Wastewater by Electrodialysis Coupled with Upflow Anaerobic Sludge Blanket and Membrane Bioreactor

**DOI:** 10.3390/membranes13050527

**Published:** 2023-05-19

**Authors:** Shichang Xu, Han Zhao, Lixin Xie, Keqiang Wang, Wen Zhang

**Affiliations:** School of Chemical Engineering and Technology, Tianjin Key Laboratory of Membrane Science and Desalination Technology, State Key Laboratory of Chemical Engineering (Tianjin University), Tianjin University, Tianjin 300350, China; xu_sc1@tju.edu.cn (S.X.); zhaohan20@tju.edu.cn (H.Z.); wkq438839400@tju.edu.cn (K.W.)

**Keywords:** electrodialysis (ED), upflow anaerobic sludge blanket (UASB), membrane bioreactor (MBR), chemical oxygen demand (COD), refined sugar wastewater (RSW)

## Abstract

In this paper, refined sugar wastewater (RSW) is treated by electrodialysis (ED) coupled with an upflow anaerobic sludge blanket (UASB) and membrane bioreactor (MBR). The salt in RSW was first removed by ED, and then the remaining organic components in RSW were degraded by a combined UASB and MBR system. In the batch operation of ED, the RSW was desalinated to a certain level (conductivity < 6 mS·cm^−1^) at different dilute to concentrated stream volume ratios (V_D_/V_C_). At the volume ratio of 5:1, the salt migration rate *J_R_* and COD migration rate *J_COD_* were 283.9 g·h^−1^·m^−2^ and 13.84 g·h^−1^·m^−2^, respectively, and the separation factor *α* (defined as *J_COD_/J_R_*) reached a minimum value of 0.0487. The ion exchange capacity (*IEC*) of ion exchange membranes (IEMs) after 5 months of usage showed a slight change from 2.3 mmol·g^−1^ to 1.8 mmol·g^−1^. After the ED treatment, the effluent from the tank of the dilute stream was introduced into the combined UASB-MBR system. In the stabilization stage, the average COD of UASB effluent was 2048 mg·L^−1^, and the effluent COD of MBR was maintained below 44–69 mg·L^−1^, which met the discharge standard of water contaminants for the sugar industry. The coupled method reported here provides a viable idea and an effective reference for treating RSW and other similar industrial wastewaters with high salinity and organic contents.

## 1. Introduction

Among the numerous agricultural industries, the sugar industry is one of the most common industries in many developing countries in Asia, Africa, and South America [1]. Refined sugar is the highest quality and purity sugar product, which is made from raw sugar that has been chemically purified, recrystallized, and boiled [2]. Since the color standard of refined sugar is tougher than that of raw sugar, the decolorization process is a key part of the purification process. Refined sugar wastewater (RSW) mainly comes from the purification process, in which a large amount of brine is used to regenerate ion exchange resins [3]. As a result, the RSW usually has a high salinity and high level of organic contaminants.

Some physicochemical methods have been applied to treat sugar industry wastewater, including electrochemical [4], electroflocculation [5], catalytic thermal treatment [6], adsorption [7], advanced oxidation [8], etc. However, these methods have drawbacks in terms of high energy input, usage of a large amount of chemical reagents, and generation of toxic by-products [3]. Biological removal of wastewater, as a greener and more sustainable approach, is attracting increasing attention. It has the characteristics of low cost, high removal rate of organic matter, and good environmental sustainability [3]. The high contents of carbohydrates in RSW endow it with a good biodegradability. However, excessive inorganic salt content in wastewater can severely inhibit microbial activity, resulting in poor biological treatment efficiency [9]. Chen et al. [10] studied the effect of salinity on removal performance using activated sludge characteristics and microbial communities in sequencing batch reactors, in which the removal rate of chemical oxygen demand (COD) decreased significantly from 94.88% to 55.78% when the salinity increased from 0 to 20 g·L^−1^ (2 wt.%). Therefore, removing salts in wastewater before biological treatment may be an effective treatment method.

Electrodialysis (ED) is an electrically driven membrane process in which the ions in solution are removed or concentrated due to the selectivity of the ion exchange membranes (IEMs). Currently, ED has been applied to the desalination of various high-salinity organic wastewaters [11,12,13,14], such as dairy wastes [15,16,17], brackish water [18], municipal wastewater [19], greenhouse wastewater [20], etc. For example, Luiz et al. [21] applied laboratory-scale ED to the desalination of sugar cane juice (conductivity of 3.2 mS·cm^−1^), which has a similar composition as RSW, and removed the salts completely within a treatment time of 1 h.

Anaerobic digestion, as a widely used treatment method for wastewater with high organic content, has the merits of energy production, low sludge yield, and low energy consumption (no consumption of aeration power). The upflow anaerobic sludge blanket (UASB) is the most widely used high-efficiency anaerobic bioreactor. Goodwin et al. [22] used a pilot-scale UASB reactor to treat canned malt liquor. A hydraulic retention time (HRT) of 2.1 d and an organic loading rate of 5.46 kgCOD·m^−3^·d^−1^ were obtained for stable operation, and a COD removal efficiency of more than 80% was achieved. However, a common downside of anaerobic treatment technologies is the incomplete removal of organic matter. In order to comply with the strict discharge regulations, coupling anaerobic digestion with aerobic MBR technology is a feasible strategy to eliminate the organic matter and obtain a high-quality effluent.

In this work, the method of ED coupled with a UASB-MBR system was used to treat RSW, in which ED was first used to reduce the salinity of RSW below a specific level and then the UASB-MBR system was used to degrade the remaining organic matter. The possibility of removing salt from RSW with a smaller amount of concentrated solution was explored by varying the volume ratios of dilute and concentrated streams in batch mode. The migration of organics in the desalination process was explored. In addition, the performance of IEMs after five months of use was tested. A UASB-MBR system was adopted to organically digest the ED dilute stream effluent and the removal of COD was examined.

## 2. Experiment

### 2.1. Wastewater

The refined sugar wastewater (RSW) in this study was from a refined sugar factory and the composition of RSW is listed in Table 1 as the raw wastewater. In order to remove solid particles to meet the influent demand of ED, pretreatments of coagulation and filtration were carried out. As the coagulant, 10 g·L^−1^ aluminum ferric chloride (basic, industrial grade) and 0.15 g·L^−1^ polyacrylamide (analytical grade) were used. The supernatant after coagulation was separated by microfiltration (0.45 μm), and the composition of filtrate is also shown in Table 1.

### 2.2. ED Setup

**Figure 1:**
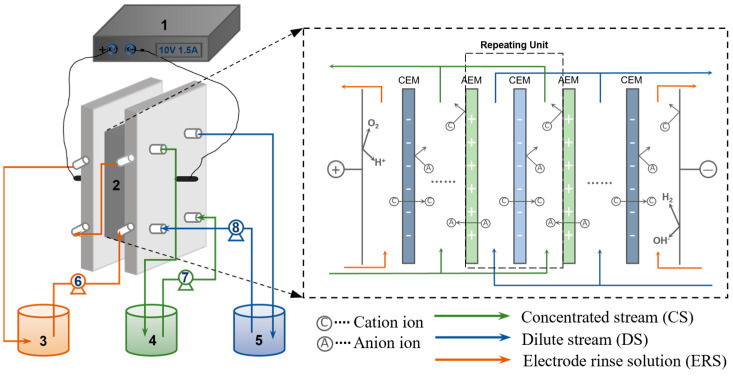
Schematic electrodialysis (ED) setup containing (1) a DC power supply, (2) a membrane stack with ten repeating units, (3) a tank of ERS, (4) a tank of CS, (5) a tank of DS, and (6–8) three circulating pumps.

The laboratory-scale ED configuration consisted of a DC power supply, an ED membrane stack, three circulatory pumps, and three tanks, which were assembled as shown in the schematic diagram in Figure 1. The ED membrane stack consisted of 10 cell pairs. Each cell pair consisted of a dilute compartment, a concentrated compartment, a cation exchange membrane (CEM), and an anion exchange membrane (AEM). The ion exchange membranes (IEMs) were made by Shandong China Tianwei Membrane Technology Co., Ltd., and their characteristics are shown in Table 2. Each membrane had an effective area of 0.0084 m^2^ (70 mm × 120 mm). The electrode membranes were perfluorosulfonic acid CEMs. A specifically shaped polyvinyl chloride spacer (thickness: 550 μm) separated the IEMs and guided the flow through to form the dilute and concentrated compartments. That is, the size of the membrane in the ED stack is 550 µm, the thickness of the spacer.

Experiments were conducted to explore the ED desalination process at different dilute to concentrated stream volume ratios (V_D_/V_C_). Dilute stream (DS), concentrated stream (CS), and electrode rinse solution (ERS) were independently circulated in their respective tanks with a flow rate of 30 L·h^−1^. The CS was 300 mL of deionized water (conductivity < 4 μS·cm^−1^), and the DS was designed as 300 mL, 900 mL, and 1500 mL of RSW. The electrode rinse solution (ERS) was 0.2 mol·L^−1^ Na_2_SO_4_ solution. At regular intervals, samples were taken from the tanks of dilute and concentrated streams and the conductivity was measured. The ED device current was recorded every 1~2 min. When the conductivity of DS dropped to below 6 mS·cm^−1^, the experiment was stopped and samples were taken to test the COD in the dilute and concentrated tanks. For each experiment, the stream in each tank was circulated to allow the liquid to fill the device before the operating voltage drop was applied. In order to eliminate potential bias in results caused by membrane contamination, the ED stack was rinsed successively with sodium hydroxide (1%, analytical grade), hydrochloric acid (1%, analytical grade), and deionized water after each batch of experiments. The experiments were conducted at a fixed stack voltage of 10 V as well as at atmospheric conditions and 25 ± 0.5 °C.

### 2.3. UASB and MBR System

**Figure 2:**
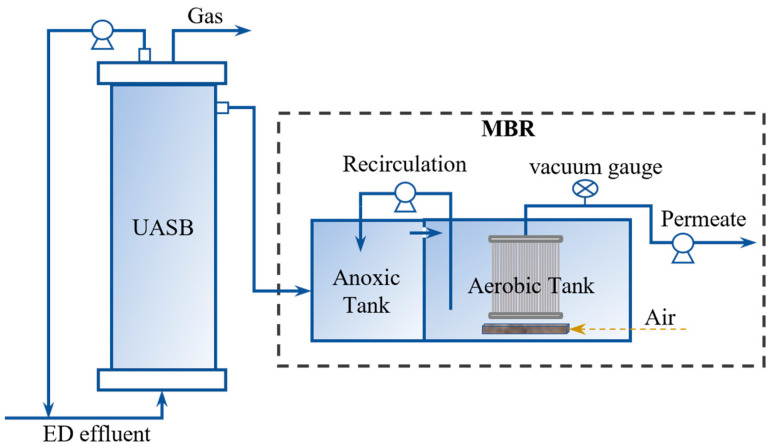
UASB-MBR process flow diagram.

This study used an upflow anaerobic sludge blanket (UASB) reactor and a membrane bioreactor (MBR) to reduce the organic matter in RSW after ED desalination. The process flow is shown in Figure 2. The UASB reactor (with a total effective volume of 10 L) was made of organic glass tubes. The effluent from the ED dilute tank was fed from the bottom of the UASB reactor at a certain flow rate (5 L·d^−1^) through a peristaltic pump. A return port was set at the top of the UASB to realize the return flow of effluent by connecting a peristaltic pump, to improve the rising flow rate of the UASB.

The inoculated sludges in the UASB and MBR were taken from the anaerobic tank and secondary sedimentation tank of a wastewater treatment plant, respectively. During the domestication stages, the feed water of the UASB was the diluted ED dilute tank effluent. To gradually domesticate the sludge, the ED dilute tank effluent was mixed with tap water at different dilution ratios before feeding to the UASB. The dilution ratio is defined as the proportion of the ED dilute tank effluent to the total feed water. Due to the lack of nutrients in the RSW, supplemental ammonium chloride, potassium hydrogen phosphate, and potassium dihydrogen phosphate (analytical grade, Tianjin China Jiangtian Chemical Technology Co., Ltd.) were added to it at a certain ratio (COD/N/P = 200:5:1).

The MBR consisted of an anoxic tank (2 L) and an aerobic tank (3 L) with an internal reflux ratio of 2:1 achieved by a circulation pump. The effluent from the top of the UASB was introduced into the anoxic tank and a stirrer was used to achieve uniform mixing of the substrate in the anoxic tank. The hollow fiber microfiltration membrane module was directly immersed in the aerobic tank. It was made of polyvinylidene fluoride (PVDF) with a membrane pore size of 0.1 μm and a total effective filtration area of 0.4 m^2^. The aeration device provided a suitable dissolved oxygen (DO) concentration (2–4 mg·L^−1^) for the aerobic tank. A peristaltic pump was used to collect membrane module permeate at a constant flux, and a vacuum gauge was connected between the two to monitor transmembrane pressure (TMP). When the TMP exceeded 0.05 MPa, the membrane module was cleaned off-site, first by soaking in a 0.5% sodium hypochlorite solution for 12 h and then rinsing with water.

When the UASB completed domestication, aerobic sludge was inoculated into the aerobic tank of the MBR. Similarly, the UASB effluent was introduced into the MBR system at a certain dilution ratio, which was changed for complete domestication. Indicators, including pH and COD of the influent and effluent, were monitored regularly.

### 2.4. Analytical Methods

The conductivity was determined by a conductivity meter (DDSJ-308A; Shanghai China INESA Scientific Instruments Co., Ltd., Shanghai, China). The pH was determined by a pH meter (PHS-3C; Shanghai China Yidian Scientific Instruments Co., Ltd., Shanghai, China). The *DO* was determined by a dissolved oxygen analyzer (JPB-607A; Shanghai China INESA Scientific Instrument Co., Ltd., Shanghai, China). The COD was determined by rapid digestion spectrophotometry using a COD digestion instrument (DRB 200, HACH, Ames, IA, USA) and a visible spectrophotometer (DR 3900, HACH, USA). The ion exchange capacity (*IEC*) and transport number (*ṫ*) were tested using the method reported in the literature [23]. The area electric resistance (*R_m_*) was measured by an electrochemical system (PARSTAT 2273, Princeton). The chemical groups on the membrane surface were detected by Fourier total reflection infrared spectroscopy (FT-IR, Nicolet 6700).

### 2.5. Data Analysis

The desalination rate *R*, (%) was calculated by Equation (1):

(1) $$R=\left(1-\frac{c_{t}}{c_{0}}\right)\times 100\%$$

where *c*_0_ and *c_t_* are the NaCl concentrations (g·L^−1^) in the tank of the dilute stream at the initial and termination moment, respectively. The NaCl concentration was calculated from the conductivity according to the standard curve of NaCl solution.

The salt migration rate *J_R_* (g·h^−1^·m^−2^) was calculated by Equation (2):

(2) $$J_{R}=\frac{V_{0}c_{0}-V_{t}c_{t}}{\mathrm{AN}t}$$

where *V_0_* and *V_t_* are the volume (L) of the dilute tank at the initial and termination moment, respectively, A is the effective area (m^2^) of each membrane, N is the number of cell pairs (10 pairs), *t* is the operation time (h).

The organic migration rate *J_COD_* (g·h^−1^·m^−2^) was calculated by Equation (3):

(3) $$J_{COD}=\frac{V_{t}COD_{t}}{1000\mathrm{AN}t}$$

where *COD_t_* is the COD concentrations (mg·L^−1^) in the concentrated tank at the termination moment.

The specific power consumption *E* (kWh·m^−3^) was calculated by Equation (4):

(4) $$E=\frac{\int_{0}^{t}UIdt}{V_{0}}$$

where *U* is the ED stack voltage (V), *I* is the current (A) during operation.

The current efficiency *η* (%) was calculated by Equation (5):

(5) $$\eta =\frac{\mathrm{zF}\left(V_{0}c_{0}-V_{t}c_{t}\right)}{\mathrm{NM}\int_{0}^{t}Idt}\times 100\%$$

where z is the number of ionic charges, F is the Faraday constant (96,485 C·mol^−1^), M is the molar mass of NaCl (58.44 g·mol^−1^).

## 3. Results and Discussion

### 3.1. Batch Experiments of ED

We investigated the ED desalination process of RSW in batch mode with different V_D_/V_C_ ratios. This parameter was studied mainly to explore the possibility of drawing salts from the DS with a small amount of concentrate. A higher volume ratio V_D_/V_C_ means a smaller amount of concentrate, which would be much more beneficial for its subsequent treatment. The DS was gradually increased from 300 mL to 1500 mL, while the concentrated stream was fixed at 300 mL, enabling the V_D_/V_C_ ratios from 1:1 to 5:1. The variation of treatment time and ED performance parameters at different volume ratios under a fixed desalination task was investigated.

**Figure 3:**
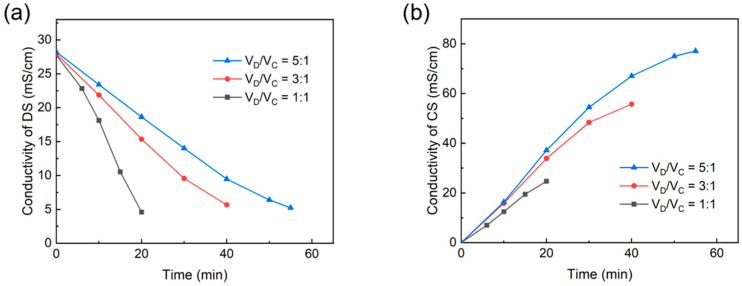
Changes in the conductivity in the (**a**) DS tank and (**b**) CS tank at different volume ratios (V_D_/V_C_).

Figure 3 shows the variation of conductivity with time, indicating a regular decrease and increase in DS and CS concentrations, respectively. The ED experiments were terminated by reducing the dilute tank effluent concentration below 6 mS·cm^−1^. Generally speaking, the salt content of sugar wastewater that can be directly biochemically treated is about 3000 mg·L^−1^ [24]. Therefore, we desalted the RSW to a uniform level of 6 mS·cm^−1^ (about 3000 mg·L^−1^ NaCl). As the V_D_/V_C_ changed from 1:1 to 5:1, the total desalination task increased due to the increasing volume of the dilute stream, with a corresponding increase in treatment time from 20 to 55 min. However, the increase was not exactly linear. This is because, when the volume ratio was relatively small, the resistance of the two streams was the dominant factor affecting the desalination. When the volume ratio was relatively large, the reverse concentration diffusion effect weakened the positive effect caused by the increase in conductivity. When V_D_/V_C_ increased from 1:1 to 3:1, the salt concentration of CS at the termination moment increased substantially from 1.49% to 3.62%. When V_D_/V_C_ continued to increase to 5:1, the CS at the termination moment increased slightly to 5.26% due to the reverse concentration diffusion for a longer time.

**Figure 4:**
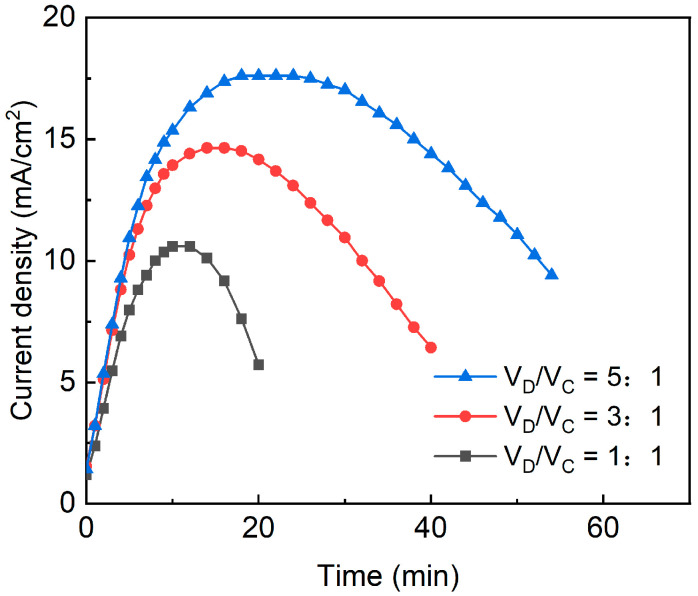
Changes in current density over time at different volume ratios (V_D_/V_C_).

The variation of the current density with time is shown in Figure 4, from which it can be observed that the current density always showed a trend of first increasing and then decreasing with time for different volume ratios. This is caused by changes in the dominant part of the resistance of the ED stack. The deionized water used as a draw solution in the early stage and the low-salinity wastewater in the later dilution zone successively have a high contribution to the total resistance. This point is also exhibited in Figure 3. At larger volume ratios, the current density was generally at a higher level. When V_D_/V_C_ was 1:1, the total amount of ions in the dilute tank was smaller, and therefore its concentration dropped faster and the total current of the device was lower. In ED processes, when the concentrations of CS and DS are nearly equal, the current will reach its maximum. Hence, with the increase in V_D_/V_C_ ratios, the time to reach the maximum of currents became later in Figure 3.

We calculated the average current, current efficiency, and specific energy consumption of the ED desalination process. From Table 3, the average current increased from 0.6830 A to 1.1900 A when the volume ratio increased from 1:1 to 5:1, which corresponds to Figure 4. The energy consumption was directly related to the current, but the trend of specific energy consumption did not exactly coincide with the current due to the simultaneous increase in the desalination. The specific energy consumption remains basically at the same level when the volume ratio changes from 1:1 to 5:1. This suggests that it is technically feasible to use a higher volume ratio V_D_/V_C_, i.e., a relatively smaller amount of CS, to extract the salts from the wastewater.

The slight decrease in current efficiency at the volume ratio of 5:1 can be attributed to the increase in concentration difference enhancing the back diffusion of salts from the concentrated compartment to the dilute compartment. However, the current efficiency always remained at a higher level above 90%, which was excellent compared to the related literature in which performance similar to ED wastewater desalination was reported [25]. The reason may be, on the one hand, that the organic components in RSW are mainly small molecules of sucrose and other carbohydrates, both of which are not charged. Therefore, the applied electric field was mainly used to remove inorganic salts. On the other hand, the ED operation was in the suitable voltage range (below limiting current density) so that no abnormalities such as ion “depletion” at the membrane surface occurred [12]. In addition, the high level of current efficiency also benefited from the improvement of mass transfer by the mesh spacers used, which prevented the concentration polarization [26,27].

**Figure 5:**
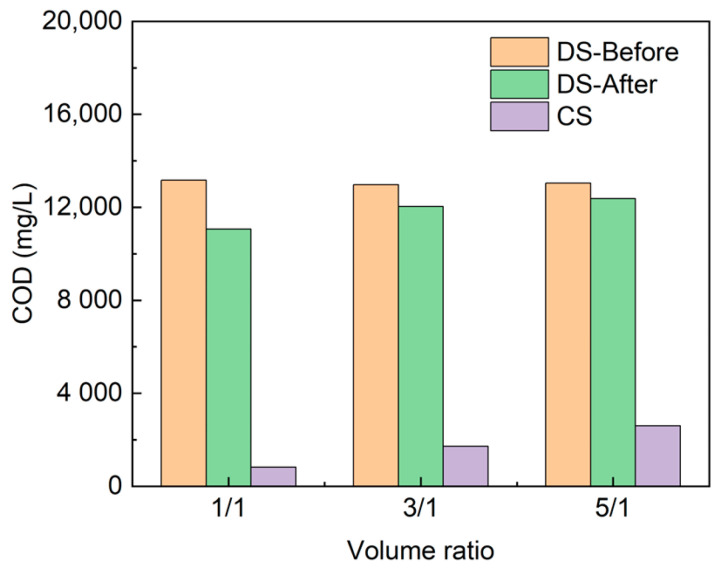
Changes in COD before and after desalination at different volume ratios (V_D_/V_C_), including DS before and after experiments (DS-Before, DS-After), CS.

A detailed examination of the change in the organic content of the dilute and concentrated tank before and after desalination is presented in Figure 5. As V_D_/V_C_ increased, the organic content in the tank of CS at the end point increased linearly from 820 mg·L^−1^ to 2600 mg·L^−1^ due to the increase in treatment time. However, the change in the COD concentration of DS before and after the ED experiments gradually became smaller as the volume ratio V_D_/V_C_ increased. This is probably because the migration of various hydrated ions during the desalination process and other “water escape” phenomena caused the dilute stream to be concentrated. Small parts of organic substances are deposited on membranes. For example, at a volume ratio of 5:1, after calculation, 95.05% COD in the initial DS tank is retained in the DS, and 3.97% is transferred to the CS tank. Therefore, approximately 1% of COD deposits on the membrane surface.

**Figure 6:**
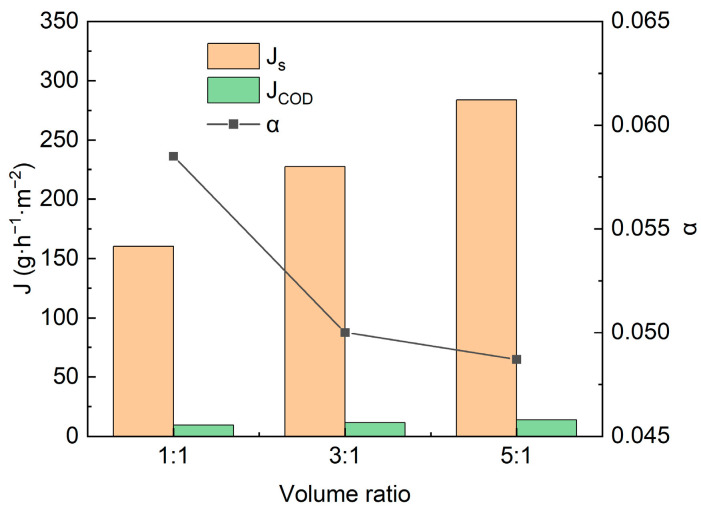
Salt migration rate (*J_s_*), COD migration rate (*J_COD_*), and the ratio *α* (=*J_COD_/J_s_*) at different volume ratios (V_D_/V_C_).

In order to better compare salt and organic migration during ED, the separation factor α was defined as the ratio of COD migration rate *J_COD_* to salt migration rate *J_s_*, i.e., *α* = *J_COD_/J_S_*. As can be seen from Figure 6, α reached a minimum of 0.0487 at a volume ratio of 5:1. For the migration of organics, there are two possible mechanisms: first, the diffusion of organics across the membrane, and second, the electrical migration of charged small molecule organics [28]. The results of this experiment showed that electromigration may be the main mechanism of organic matter migration, which was consistent with that reported in the literature [21]. The point was also confirmed in the diffusion experiments driven by concentration difference, which showed that the organic migration rate in the absence of an external electric field was 0.6 g·h^−1^·m^−2^, much lower than that in the electric field-driven case.

Irrespective of the organic migration mechanism, at a volume ratio V_D_/V_C_ of 5:1, for RSW, ED accomplished a desalination rate of up to 83.5% while the organic matter loss was only 4.95%. The results of the present experiment may demonstrate that the desalination of the RSW by ED is technically feasible. Meanwhile, in the actual production, the suitable volume ratio range of V_D_/V_C_ can be selected with reference to the results of this experiment to ensure the desalination effect and reduce the loss of organic matter at the same time.

### 3.2. Changes in the Properties of IEMs

We verified the stability of IEMs in a five-month operation. IEMs were treated with 1 mol·L^−1^ HCl and 1 mol·L^−1^ NaOH and then washed to neutral before testing. The results are shown in Table 4. The transport number (*ṫ*) reflects the selective permeability of the IEMs and remains basically unchanged before and after the operation. The ion exchange capacity (*IEC*) value and area electric resistance (*R_m_*) are related to the anti-ion exchange ability and conductivity of the IEMs, respectively. The decrease in *IEC* values and the increase in *R_m_* reflect slight membrane fouling on IEMs, but it is still within an acceptable range. Overall, the RSW system did not damage the IEMs, and their performance remains relatively stable after use for five months. 

**Figure 7:**
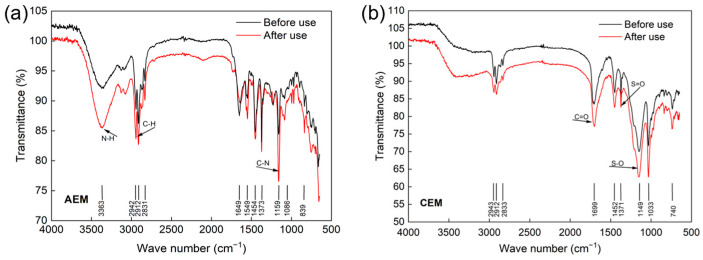
FT-IR images of AEM (**a**) and CEM (**b**).

The FT-IR spectra of IEMs before and after 5 months of usage were tested in the range of 500–4000 cm^−1^. From Figure 7a, the stretching vibrational absorption peaks at 3363 cm^−1^ and 1159 cm^−1^ belong to N-H and -C-N- groups, respectively. A distinct absorption peak at 1149 cm^−1^ is observed in Figure 7b, which may be derived from the -S-O- vibration of the sulfonic acid group. In Figure 7, no significant new peaks appeared in the FT-IR spectra of IEMs after use, compared to the fresh membranes. Overall, the IEMs maintained a good stability in the process of ED desalination of RSW.

### 3.3. Biodegradation of ED Dilute Stream Effluent

A UASB-MBR system was used for biochemical degradation of the dilute effluent from ED (salinity < 0.3%). The whole experiment lasted for 150 d, and the influent organic loading rate (OLR) at each stage is shown in Table 5. Through eight phases of progressive influent, the OLR gradually increased from 0.65 kgCOD·m^−3^·d^−1^ to 6.5 kgCOD·m^−3^·d^−1^. 

**Figure 8:**
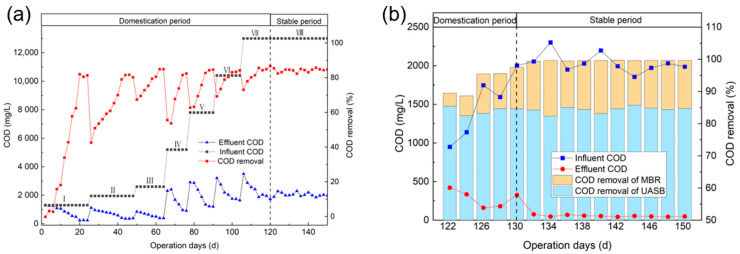
Influent and effluent COD and COD removal efficiency by UASB (**a**) and MBR (**b**).

The removal of COD by the UASB in each stage is shown in Figure 8a. Ⅰ-Ⅷcorrespond to each stage in Table 5. It can be found that, in the first 10 days, a lower COD removal efficiency (less than 20%) was obtained, reflecting the insufficient microbial regulation caused by environmental changes during the initiation stage of the UASB. When the removal rate reached about 80% at each stage, the OLR of feed water was increased and the domestication moved into the next phase. For each stage, the total removal rate of COD showed a similar tendency of first decreasing and then increasing, because the sudden lifting of the load could have a certain negative impact on the system. However, the COD removal rate can usually become stable in about 10 days. Over time, the microorganisms gradually adapted to the water quality and began to rapidly propagate, so the removal rate was also greatly enhanced. Despite the continuously increasing OLR, the COD removal rate of the UASB steadily increased and eventually stabilized at about 85.1%, with an average COD value of 2048 mg·L^−1^ in the effluent.

In the 8th stage, the UASB effluent was diluted to a certain ratio and introduced into the MBR for further degradation. The HRT of the MBR was 1 day and the DO concentration in its aerobic tank was maintained at 2–4 mg·L^−1^ by adjusting the aeration. As shown in Figure 8b, the contribution of the MBR to organic matter degradation gradually increased during the 10-day domestication process. Finally, the combined UASB-MBR system achieved a total COD removal of up to 99.6%, in which the MBR contributed a share of about 15.1%. Finally, the combined UASB-MBR system achieved a total COD removal of up to 99.6%. In the stable stage, the COD of the effluent from the MBR aerobic tank remained at 44–69 mg·L^−1^, which can meet the standard of the Discharge Standard of Water Contaminants for Sugar Industry (GB 21909-2008) [29].

The main cost of the ED process is the power consumption for desalting, which is calculated to be about 7 kWh·m^−3^ here. However, the overall economic costs still need to be estimated compared with other methods, such as using halophilic bacteria to treat the RSW directly. In addition, the engineering feasibility of the combined ED and MBR processes could also be investigated in future work.

## 4. Conclusions

In this work, a combined process of electrodialysis coupled with a UASB and MBR was employed for the treatment of RSW. The conclusions are as follows. ED was used to desalinate RSW to below 6 mS·cm^−1^. At a volume ratio of 5:1, the desalination rate was up to 83.5% while the organic matter loss was only 4.95%. Meanwhile, separation factor α of organic matter and inorganic salts (*J_COD_/J_s_*) reached a minimum of 0.0487. The IEMs kept their performance during 5 months of operation in ED. After 5 months of usage, *IEC* values of the IEMs slightly decreased, and there was no significant change in the ionic migration numbers *ṫ*. In the stabilization stage, the UASB and MBR contributed 84.5% and 15.1% of the total COD removal, respectively. The effluent COD of the UASB-MBR system was finally maintained at 44–69 mg·L^−1^, meeting the discharge standard of water contaminants for the sugar industry. The combined process achieved effective removal of inorganic salts and organic compounds from RSW. The effluent from this process can be reused as rinse water in other parts of the plant, which can greatly improve the water utilization in the refined sugar industry.

## Figures and Tables

**Table 1:** Changes in wastewater physicochemical parameters.

Parameter	pH	Conductivity (mS·cm^−1^)	COD (g·L^−1^)	NH_4_-N (mg·L^−1^)	Color (PCU)	Turbidity (NTU)	Suspended Solids (mg·L^−1^)
Raw wastewater	6.8–7.2	28–30	20–22	20–24	8700–8800	1600–1700	480–520
Filtrate	6.8–7.2	27–29	12–14	15–18	850–900	7–9	1–2

**Table 2:** Main performance parameters of the IEMs.

Performance	CEM	AEM
Ion exchange capacity/mmol·g^−1^	0.90~1.10	0.90~1.10
Area electric resistance/Ω·cm²	≤4.50	≤5.00
Thickness (wet)/μm	40~50	40~50
Uptake in H_2_O at 25 °C/wt%	15~20	15~20
Transport number	≥0.98	≥0.98
Stability/ph	1~12	0~14
Temperature/°C	15~40	15~40

**Table 3:** Average current ($\bar{I}$), current efficiency (*η*), specific energy consumption (*E*) of the ED desalination experiment for the RSW at different volume ratios (V_D_/V_C_).

Volume Ratio	1:1	3:1	5:1
$\bar{I}$/A	0.6830	0.9456	1.1900
*η*/% ^b^	92.13	93.34	92.51
*E*/(kW·h·m^−3^) ^a^	7.34	6.93	7.22

^a^ Calculated by Equation (4). ^b^ Calculated by Equation (5).

**Table 4:** The ED performance of AEM and CEM before and after 5 months of usage.

Performance	AEM		CEM	
	Before	After	Before	After
*IEC* (mmol·g^−1^)	2.28	1.83	2.19	2.13
*Rm* (Ω·m^−2^)	1.05	1.37	1.06	1.21
*ṫ*	0.92	0.92	0.93	0.92

**Table 5:** UASB phase description.

Phase	Dilution Ratio	Operation Periods	Organic Loading Rate (kgCOD·m^−3^·d^−1^)	HRT of UASB/d
1	10%	Days 1 to 24	0.65	2
2	15%	Days 25 to 48	0.98	2
3	20%	Days 49 to 64	1.3	2
4	40%	Days 65 to 76	2.6	2
5	60%	Days 77 to 90	3.9	2
6	80%	Days 91 to 104	5.4	2
7	100%	Days 105 to 120	6.5	2
8	100%	Days 121 to 150	6.5	2

## Data Availability

Not applicable.

## Abbreviations

AEM	Anion Exchange Membrane
CEM	Cation Exchange Membrane
COD	Chemical Oxygen Demand
CS	Concentrated Stream
DO	Dissolved Oxygen
DS	Dilute Stream
ED	Electrodialysis
ERS	Electrode Rinse Solution
HRT	Hydraulic Retention Time
IEC	Ion Exchange Capacity
IEMs	Ion Exchange Membranes
MBR	Membrane Bioreactor
OLR	Organic Loading Rate
PVDF	Polyvinylidene Fluoride
RSW	Refined Sugar Wastewater
TMP	Transmembrane Pressure
UASB	Upflow Anaerobic Sludge Blanket
